# Efficacy of early enteral nutrition in patients with traumatic brain injury: a systematic review and meta-analysis

**DOI:** 10.3389/fnut.2026.1795734

**Published:** 2026-07-23

**Authors:** Wei Zhang, Yan Lei, Qiu Lin, Xiao-Ying Yan, Hui-Bin Huang, Wen-He Zheng

**Affiliations:** 1 Department of Critical Care Medicine, The Second People’s Hospital Affiliated to Fujian University of Traditional Chinese Medicine, Fuzhou, China; 2 Department of Critical Care Medicine, Guang’anmen Hospital, China Academy of Chinese Medical Sciences, Beijing, China

**Keywords:** complication, early enteral nutrition, meta-analysis, mortality, traumatic brain injury

## Abstract

**Background:**

Severe traumatic brain injury (TBI) induces a hypermetabolic state that heightens the risk of mortality and poor neurological outcomes. Early enteral nutrition (EEN), typically defined as initiation within 48 h of injury, is considered crucial, yet robust evidence of its specific benefits in TBI patients remains limited.

**Methods:**

Following PRISMA guidelines, we systematically searched the PubMed, Embase, Web of Science, and Cochrane Library databases through December 25, 2025, for studies comparing EEN with delayed enteral nutrition (DEN) in adults with TBI. Outcomes included clinical outcomes, nutrition status, and complications. Study quality was assessed using the Newcastle-Ottawa Scale and the Cochrane Risk of Bias tool. The primary outcome was short-term mortality, defined as from hospital admission to 90 days post-discharge. Sensitivity analyses and subgroup analyses explored heterogeneity.

**Results:**

Ten studies (6 randomized controlled trials [RCTs]; 4 observational; 13,046 patients) were included. When all studies (RCTs and observational) were pooled, there was no significant difference in short-term mortality risk between EEN and DEN (risk ratio [RR] = 0.64; 95% CI, 0.32–1.31). However, a prespecified sensitivity analysis limited to RCTs showed a significant reduction in mortality with EEN (RR = 0.58; 95% CI, 0.35–0.96). EEN significantly shortened mechanical ventilation duration and reduced both ICU and hospital length of stay. Nutritional status, including serum albumin levels, glucose control, caloric intake, and positive nitrogen balance, was markedly improved with EEN, without increasing hospitalization costs. EEN also significantly lowered rates of sepsis, central nervous system infection, stress ulcer, ventilator-associated pneumonia, and total adverse events (all *p* values range from <0.00001 to 0.04), though it was associated with a mildly elevated risk of diarrhea (*p* = 0.02).

**Conclusion:**

In this meta-analysis, EEN initiated within 48 h of TBI showed a trend toward reduced short-term mortality and improvements in clinical and nutritional outcomes compared to DEN. However, these findings should be interpreted cautiously given the high risk of bias and substantial statistical heterogeneity in the included studies. Future high-quality, large-scale RCTs are needed to confirm these effects and refine optimal feeding strategies in this population.

**Systematic review registration:**

https://inplasy.com/wp-content/uploads/2026/01/INPLASY-Protocol-8729.pdf.

## Introduction

Severe traumatic brain injury (TBI) is a leading cause of high mortality and long-term disability, with approximately 40% of patients dying from their injuries and around 60% experiencing poor neurological outcomes (1, 2). This not only imposes a substantial caregiving burden but also exerts a heavy economic toll on patients and healthcare systems (3). TBI triggers a hypermetabolic state characterized by elevated energy expenditure, accelerated protein catabolism, muscle wasting, and hypoalbuminemia, which impairs immune function and increases susceptibility to hospital-acquired infections (4–7). Retrospective studies have consistently shown that delayed initiation of nutritional support after injury is independently associated with higher mortality rates (8), underscoring the critical role of timely nutritional intervention in the management of severe TBI.

Early enteral nutrition (EEN), typically initiated within 24 to 48 h post-injury, has been shown to improve clinical outcomes, including reduced infection rates, shorter hospital stays, and potentially decreased mortality (8–10). Moreover, EEN may help attenuate the catabolic and inflammatory processes specific to TBI (11). Despite these potential benefits, clinical implementation of EEN remains challenging due to several concerns, such as the risk of aspiration pneumonia, transient elevations in intracranial pressure during feeding tube placement (12, 13), and frequent delays in gastric emptying (14). These factors often lead to the postponement of enteral nutrition (EN) in clinical practice.

Although major clinical guidelines, including those from the European Society of Intensive Care Medicine and the fourth edition of the Brain Trauma Foundation Guidelines, advocate for EEN largely based on expert consensus (15, 16), high-quality evidence remains limited. Existing randomized controlled trials (RCTs) are generally small in scale and underpowered (17–20). A recent meta-analysis comparing EEN (initiation within 48 h) with early parenteral nutrition (PN) or delayed EN comprised a heterogeneous population of critically ill patients (16). Notably, it included only three small studies involving TBI patients and failed to demonstrate a clear advantage in reducing mortality or pneumonia incidence (16). Additionally, this analysis did not evaluate other clinically relevant endpoints, such as nutrition status, duration of mechanical ventilation, or complications.

To address this evidence gap, we conducted a meta-analysis of patients with TBI, comparing clinical outcomes between early and late EN. We hypothesized that EEN would be associated with improved patient prognosis. The findings from this analysis are expected to inform the development of evidence-based practice guidelines and optimize clinical care for this patient population.

## Methods

### Reporting standards and registration

This systematic review followed the PRISMA 2020 Statement (Preferred Reporting Items for Systematic Reviews and Meta-Analyses) (21) (see Supplemental File 1). The study protocol was registered with the International Platform of Registered Systematic Review and Meta-analysis Protocols (Registration ID: INPLASY202610081) before initiating data extraction.

### Search strategy

An extensive literature search was conducted through December 25, 2025, across four databases: PubMed, Embase, Cochrane Library, and Web of Science. Search main terms included (traumatic brain injury OR craniocerebral injury OR head injury OR head trauma) AND (enteral nutrition OR enteral tube feeding OR feeding OR parenteral nutrition OR gastrointestinal OR intubation OR nutritional support). No language or sample size restrictions were imposed. Additionally, grey literature sources, such as ClinicalTrials.gov, medRxiv, and Google Scholar, were also systematically reviewed. Reference lists of included articles and previous meta-analyses were manually examined to identify additional eligible studies. The complete search strategy is available in Supplemental File 2.

### Inclusion and exclusion criteria

The inclusion criteria were guided by the PICOS framework:

1. Participants: Adults (≥18 years) with confirmed TBI.
2. Intervention: Initiation of EN within 48 h of injury, regardless of combined PN therapy, nutrition route, and EN formula.
3. Comparator: Initiation of EN at least 48 h after injury, regardless of PN combination, nutrition route, and formula.
4. Outcomes:

Clinical outcome: Primary outcome was short-term mortality (defined as all-cause mortality from admission to 90 days post-discharge). Secondary outcomes were length of stay (LOS) in ICU and hospital, duration of mechanical ventilation (MV), and hospitalization cost.

Nutrition status: Serum albumin levels, control of blood glucose levels, nitrogen balance, and other indicators after treatment (defined by study authors).

Complication: Total adverse events, various nosocomial infections, stress ulcer, diarrhea, and others, if reported.

5 Study Design: RCTs, cohort studies (prospective/retrospective), and case–control studies.

Exclusion criteria: Pediatric studies, animal or cell model studies, review articles, single-arm trials without comparators, and abstracts lacking full data. Studies that defined EEN as 48 h outside of TBI or had an unclear definition of EEN (e.g., recovery of intestinal tolerance) were also excluded.

### Literature selection and data extraction

EndNote X9 was used for literature management. Two reviewers (W-HZ and D-XY) independently screened titles, abstracts, and full texts, resolving discrepancies through consensus or consultation with a third reviewer (H-BH). Data were extracted using pre-piloted Excel forms, capturing:

(1) Patient demographics: Country, age, sex, body mass index, TBI severity defined by Glasgow Coma Scale, and baseline nutrition status.
(2) Intervention details: EEN and DEN regimen, PN use, nutrition route, and treatment duration.
(3) Predefined outcomes and quality assessment data from the included studies. Disagreements during extraction were adjudicated by H-BH.

### Quality assessment of literature

Cohort and case–control studies were evaluated using the Newcastle-Ottawa Scale, which assesses three domains: cohort selection, group comparability, and outcome measurement (22). Studies could score up to 1 point per item in selection and outcome domains, and up to 2 points in comparability. RCTs were evaluated using the Cochrane risk of bias tool (23). Total scores (0–9) categorized studies as high (8–9), moderate (6–7), or low (<6) quality.

### Statistical analysis

A meta-analysis was conducted to assess the data-reporting formats used in the included studies. Pooled odds ratios (ORs) and mean differences (MDs) with corresponding 95% confidence intervals (CIs) were calculated for binary and continuous outcomes, respectively. Medians and IQRs were converted to means and SDs using Wan et al. (24): mean = (Q1 + Median+Q3)/3; SD = (Q3–Q1)/1.35 (*n* > 25) or (Q3–Q1)/3.3 (*n* < 25). A sensitivity analysis was conducted employing a simplified method, where the mean was approximated by the median, and the standard deviation (SD) was estimated as the interquartile range (IQR) divided by 1.35. For studies providing comparative data across groups, relationships were examined by pooling ORs from these groups.

Heterogeneity was evaluated using the *I*^2^ statistic, with results classified as low (*I*^2^ < 50%) or high (*I*^2^ > 50%). Pooled analyses were conducted using a random-effects model in the presence of significant heterogeneity, and a fixed-effects model otherwise (25). To explore potential sources of heterogeneity regarding the primary outcome, we conducted sensitivity analyses by sequentially excluding individual studies to assess their influence on the overall effect estimate. We further pooled the studies limited to (1) study design: RCTs or non-RCTs; (2) DEN type: DEN with PN or DEN without PN; (3) calorie intake: fixed target calorie, progressive advance calorie or unclear calorie intake; (4) nutritional route: nasogastric tube or unclear; and (5) TBI severity: severe or moderate to test the robustness of the outcomes. Our study was conducted using Review Manager (version 5.4). Publication bias was assessed using funnel plots and Egger’s linear regression test, performed with Stata version 19.5 (Stata Corp, College Station, TX, USA). A *p*-value below 0.05 was regarded as statistically significant.

## Results

### Trial identification and study characteristics

**Figure 1:**
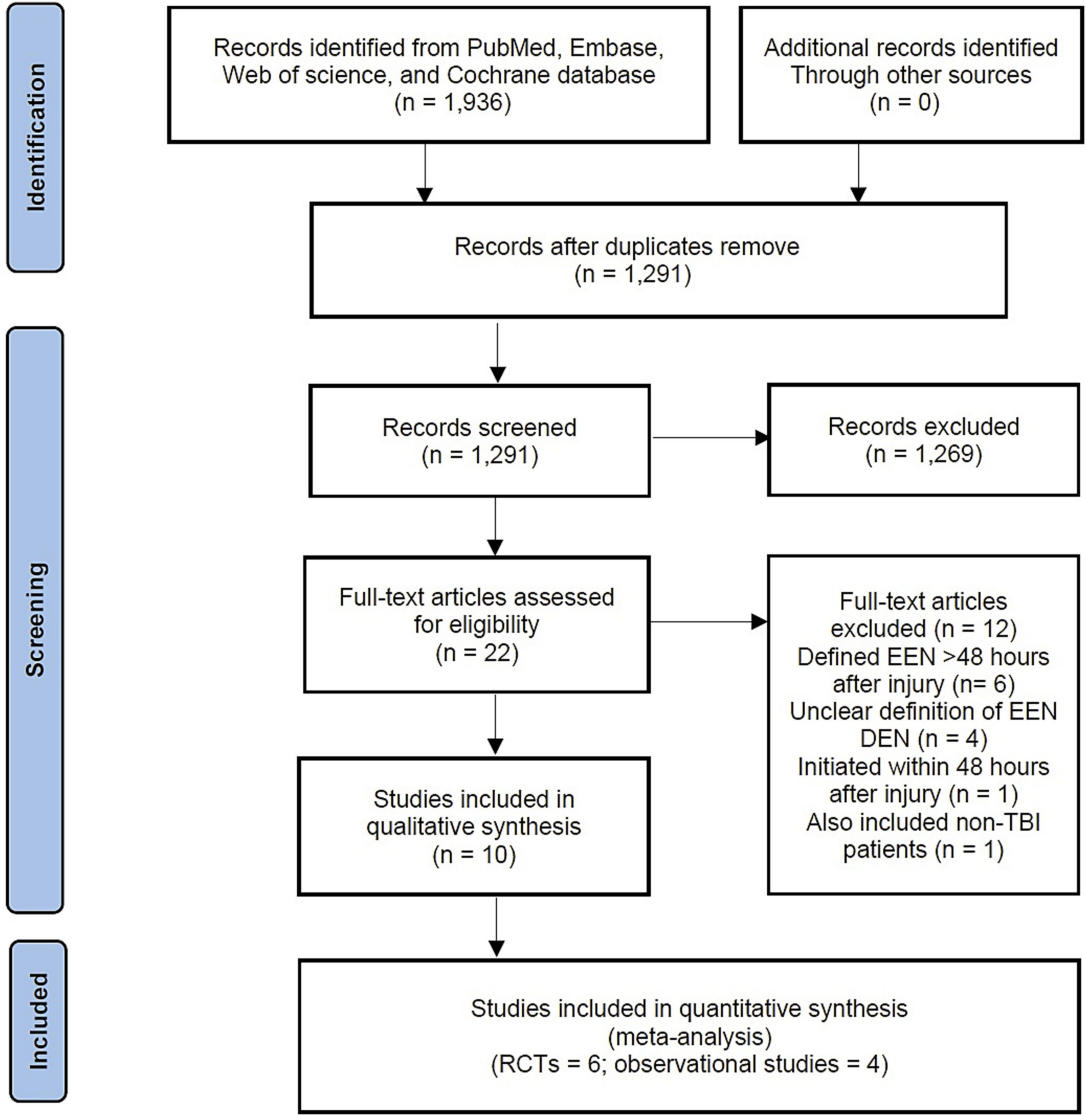
Selection process for studies included in the meta-analysis. EEN, early enteral nutrition; DEN, delayed enteral nutrition; RCT, randomized controlled trial.

The initial search yielded 1,936 entries. After eliminating duplicates and screening titles and abstracts, 22 articles were selected for full-text assessment. Among these, 12 studies were excluded based on the following criteria: defined EEN > 48 h after injury (*n* = 6); DEN initiated within 48 h after injury (*n* = 1); unclear definition of EEN (*n* = 4); and also included non-TBI patients (*n* = 1) (Supplemental File 3). Consequently, 10 studies involving 13,046 patients were deemed eligible for the present meta-analysis (10, 11, 17–19, 26–31) (Figure 1).

Key characteristics of the included studies and protocols for EEN and DEN are outlined in Table 1 and Supplemental File 4. These studies were conducted in China (*n* = 4), USA (*n* = 3), Japan (*n* = 1), Brazil (*n* = 1) and Greece (*n* = 1). Six included studies were RCTs (11, 17, 18, 27, 28, 30) and the remaining four were observational studies (10, 26, 29, 31). Most studies encompassed severe TBI (GCS ≤ 8), four focused on all types of TBI (17, 28, 29, 31), and one included moderate-to-severe TBI patients (10). Only one included study reported combination PN clearly (27). For the EEN regimen, three studies provided calories based on indirect calorimetry (11, 17, 18), and the other studies provided calories formula-based prediction methods. As to the nutritional route, three studies used nasogastric tubes, one used a nasojejunal tube, and other studies did not explicitly provide data. The GCS reported across the included studies was from 8 to 12.

**Table 1:** The study characteristics of the included studies.

Study (Year)	Country	Study design	Comparison	Sample size, n		Male, %		Mean age, years		Baseline GCS, mean		Outcomes
				EEN	DEN	EEN	DEN	EEN	DEN	EEN	DEN	
Breeding et al. (10)	USA	Retrospective, single-center	EEN vs. DEN	2,272	6,938	74.0	76.0	53.2	50.1	6.1	5.9	①, ②, ③, ④, ⑥
Chiang et al. (26)	China	Retrospective, multi-center	EEN vs. non-EN	145	152	69.0	75.7	55.0	57.0	5.6	5.6	①, ⑧
Chourdakis et al. (11)	Greece	RCT	EEN vs. DEN	34	25	76.5	84.0	36.1	33.3	5.8	5.2	④, ⑤
Fan et al. (27)	China	RCT	EEN vs. PN vs. EEN + PN	80	40	52.0	40.0	40.1	41.6	6.0–8.0	6.0–8.0	①, ②, ④, ⑤, ⑥
Graham et al. (17)	United States	RCT, single-center	Early NJT vs. delayed NGT	17	15	88.0	93.0	25.5	27.8	5.1	7.1	②, ⑤
Hadley et al. (18)	United States	RCT	EEN vs. PN	21	24	85.7	91.7	27.7	27.7	5.9	5.8	①, ④, ⑤
Li et al. (28)	China	RCT	EEN vs. PN	60	60	55.0	52.0	52.3	53.1	NR	NR	②, ④, ⑤, ⑦
Li et al. (29)	China	Retrospective, single-center	EEN + PN vs. PN	33	28	72.7	82.1	51.1	44.2	5.7	5.8	③, ④, ⑤
Meirelles et al. (30)	Brazil	RCT	EEN vs. PN	12	10	92.0	90.0	31.0	31.0	9.0	9.0	①, ②, ④, ⑤
Ohbe et al. (31)	Japan	Retrospective, database	EEN vs. DEN	1,100	1980	65.0	66.0	75.0	74.0	8.0	7.9	①, ③, ④, ⑦

DEN, delayed enteral nutrition; EEN, early enteral nutrition; GCS, Glasgow Coma Scale; NGT, nasogastric tube feeding; NJT, nasojejunal tube feeding; NR, not reported; PN, parenteral nutrition; RCT, randomized controlled trial.

① Mortality; ② ICU length of stay; ③ Hospital length of stay; ④ Complications; ⑤ Nutritional status; ⑥ Duration of mechanical ventilation; ⑦ Hospital cost.

### Study quality

Evaluation of the four studies using the Newcastle-Ottawa Scale tool found them to be moderate (29) and high quality (10, 26, 31) (Supplemental File 5). Six RCTs were evaluated using the Cochrane Risk of Bias tool (11, 17, 18, 27, 28, 30) (Supplemental File 6). In general, the included studies were high risk, mainly because they did not use blinding methods (performance bias), and most RCTs did not clearly describe allocation concealment (selection bias).

### Findings from meta-analysis

#### Clinical outcomes

**Figure 2:**
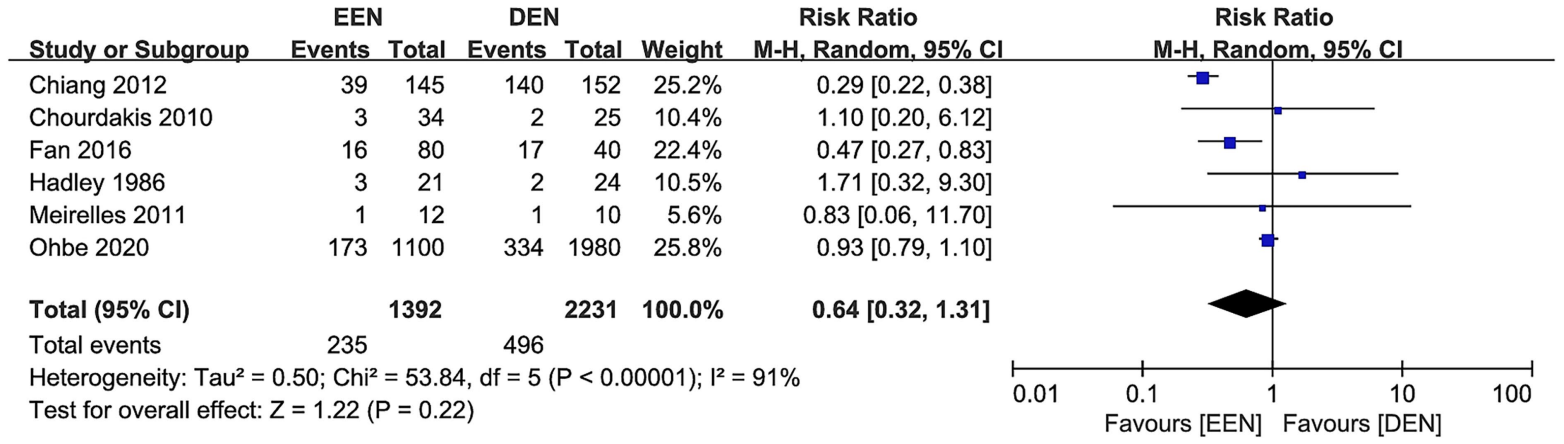
Forest plot of odds ratios for the comparisons of mortality risk between patients with TBI who received EEN and DEN. EEN, early enteral nutrition; DEN, delayed enteral nutrition; TBI, traumatic brain injury.

A total of six studies investigated the impact of EEN strategies on short-term mortality (11, 18, 26, 27, 30, 31). The pooled results (RCTs and observational) indicated that EEN had a comparable mortality risk to the DEN strategy (RR = 0.64; 95% CI, 0.32–1.31; *I*^2^ = 91%; *p* = 0.22) (Figure 2). To investigate the substantial heterogeneity, we performed comprehensive subgroup analyses based on study design, TBI severity, nutritional route, and calorie intake protocols (Table 2). The analyses revealed that study design and calorie advance strategies were the primary sources of heterogeneity. Specifically, heterogeneity was resolved (*I*^2^ = 0%) in the subgroup of RCTs using fixed-target calorie and gradually increasing calorie, with all which favored EEN. In particular, the RCT-only subgroup demonstrated a significant mortality reduction (RR = 0.58; 95% CI, 0.26–0.96; *I*^2^ = 13%; *p* = 0.03). These results suggest that study design and calorie advance strategies may be the main causes of overall heterogeneity. The test for funnel plot asymmetry for mortality was not significant (*p* = 0.703), indicating no evidence of publication bias (Supplemental File 7).

**Table 2:** Subgroup analyses of the effect of EEN on mortality in traumatic brain injury patients.

Sensitivity analysis	References	Patient number	Event in EEN Group	Event in DEN Group	Risk eatio (95% CI)	*I* ^2^	*p*
Study design: Studies of randomized controlled trials	(11, 18, 27, 30)	246	23 of 147 (15.6%)	22 of 99 (22.2%)	0.58 (0.35, 0.96)	**0%**	**0.03**
Studies of non-randomized controlled trials	(26, 31)	3,378	212 of 1,254 (16.9%)	474 of 2,133 (22.2%)	0.53 (0.17, 1.63)	98%	0.27
DEN type: Studies of EEN vs. DEN without PN	(11, 26, 31)	3,437	212 of 1,245 (17.0%)	474 of 2,133 (22.2%)	0.60 (0.22, 1.65)	96%	0.32
Studies of EEN vs. DEN with PN	(18, 27, 30)	187	20 of 113 (21.9%)	20 of 74 (27.0%)	0.58 (0.31, 1.10)	8%	0.08
TBI severity: Studies of severe traumatic brain injury	(11, 18, 26, 27, 31)	3,602	234 of 1,380 (17.0%)	495 of 2,222 (22.3%)	0.63 (0.30, 1.33)	93%	0.23
Studies of moderate traumatic brain injury	(30)	22	1 of 12 (8.3%)	1 of 10 (10%)	0.83 (0.06, 11.7)	—	0.89
Calorie intake: Study of fixed-target calorie	(27, 30)	142	17 of 92 (18.5%)	18 of 50 (36%)	0.48 (0.28, 0.84)	0%	**0.01**
Study of progressive advance calorie	(18, 31)	3,125	176 of 1,121 (15.7%)	336 of 2004 (16.8%)	0.94 (0.79, 1.11)	0%	0.45
Study of unclear calorie intake	(11, 26)	357	42 of 179 (15.7%)	142 of 177 (16.8%)	0.43 (0.13, 1.40)	56%	0.16
Nutritional route: Nasogastric tube as EN route	(11, 18, 27, 30)	246	23 of 147 (15.6%)	22 of 99 (22.2%)	0.58 [0.35, 0.96]	0%	**0.03**
Unclear EN route	(26, 31)	3,378	212 of 1,254 (16.9%)	474 of 2,133 (22.2%)	0.53 (0.17, 1.63)	98%	0.27

DEN, delayed enteral nutrition; EEN, early enteral nutrition; PN, parenteral nutrition; TBI, traumatic brain injury.

**Figure 3:**
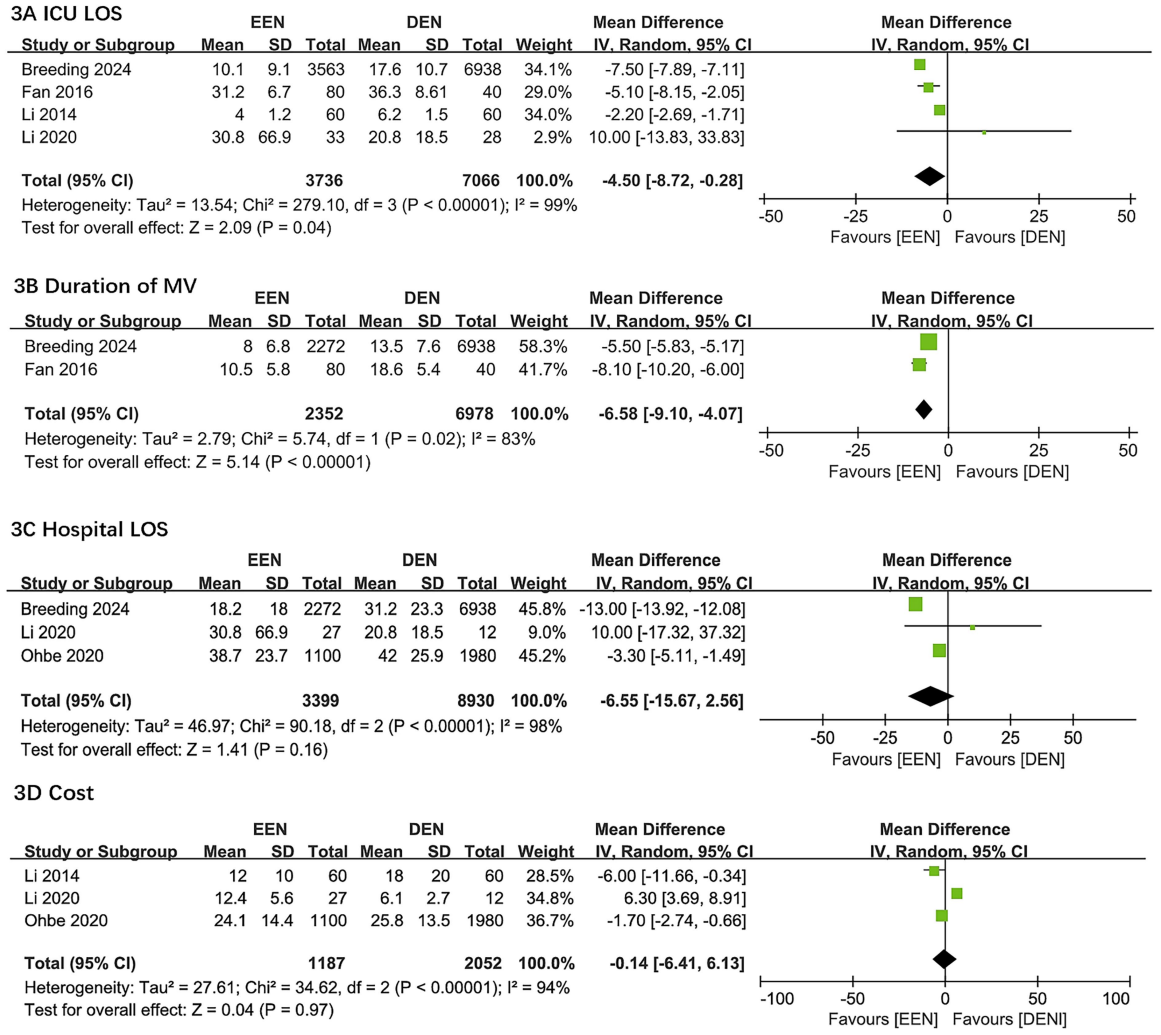
Forest plot of odds ratios for the comparisons between TBI patients with EEN and DEN regarding ICU LOS **(A)**, hospital LOS **(B)**, duration of MV **(C)** and hospitalization cost **(D)**. EEN, early enteral nutrition; DEN, delayed enteral nutrition; LOS, length of stay; TBI, traumatic brain injury; MV, mechanical ventilation.

Regarding other clinical outcomes, EEN was associated with a significant reduction in ICU LOS (10, 27–29) (MD = −4.50 days; 95% CI, −8.72 to −0.28; *p* = 0.04; Figure 3A) and duration of MV (10, 27) (MD = −6.58 days; 95% CI, −9.10 to −4.07; *p* < 0.00001; Figure 3B). Hospital LOS did not differ significantly between the EEN and DEN strategies (MD = −7.05 days; 95% CI, −15.56 to 1.47; *p* = 0.10; Figure 3C) when compared to the DEN strategy (10, 29, 31). EEN had hospitalization costs comparable to the DEN strategy (MD = −0.14; 95% CI, −6.41 to 6.13; *p* = 0.97; Figure 3D) (28, 29, 31). Additionally, three studies reported GCS changes during therapy (17, 26, 29). Chiang et al. (26) found that patients in the EEN group had significantly higher GCS scores than those in the DEN group over 7 ICU days after adjusting for age and gender (*p* < 0.001). Similarly, Li et al. (29) observed a significantly higher post-treatment GCS in the EEN group, both among patients with a GCS of 8 or lower (*p* = 0.042) and those with a GCS greater than 9 (*p* = 0.001). The study by Grahm et al. (17) indicated that the EEN group experienced greater improvement in GCS, with an average increase of 3.2, compared with 2.4 in the DEN group. In addition, For the outcomes of hospital LOS and cost, sensitivity analyses using the simplified transformation method yielded results highly consistent with our primary analysis (Supplemental File 8).

#### Nutrition status

Four studies described the intake of calories during the treatment (11, 17, 18, 30). Two studies reported that the EEN group had significantly higher caloric intake on most days of treatment than the DEN group (all *p values* <0.01) (11, 17), while another study showed a similar total calory intake between the EEN and DEN groups during 5 days (30). In the study by Grahm et al. (17), the EEN resulted in a significantly lower net caloric deficit (3,010 kcal) than the DEN group (10,194 kcal) during the first 7 days, and 45.7% (7/15) of DEN patients did not achieve full caloric demand within 7 days. In addition, Hadley et al. found that the EEN and TPN groups took 12 and 9 days to achieve target caloric delivery calories by using 140% of the basal metabolic rate.

Two studies focused on EEN vs. TPN groups and showed that, compared to the EEN group, the TPN group had significantly higher nitrogen intake via PN but equally high nitrogen loss (18, 30), resulting in a similar nitrogen balance (*p* = 0.34) (30), or an even lower proportion achieving positive nitrogen balance (1/22 vs. 4/18) (18). Another study compared EEN via nasojejunal feedings with DEN via gastric feedings, and found that the EEN group had significantly improved nitrogen intake and nitrogen balance than the DEN group (17).

**Figure 4:**
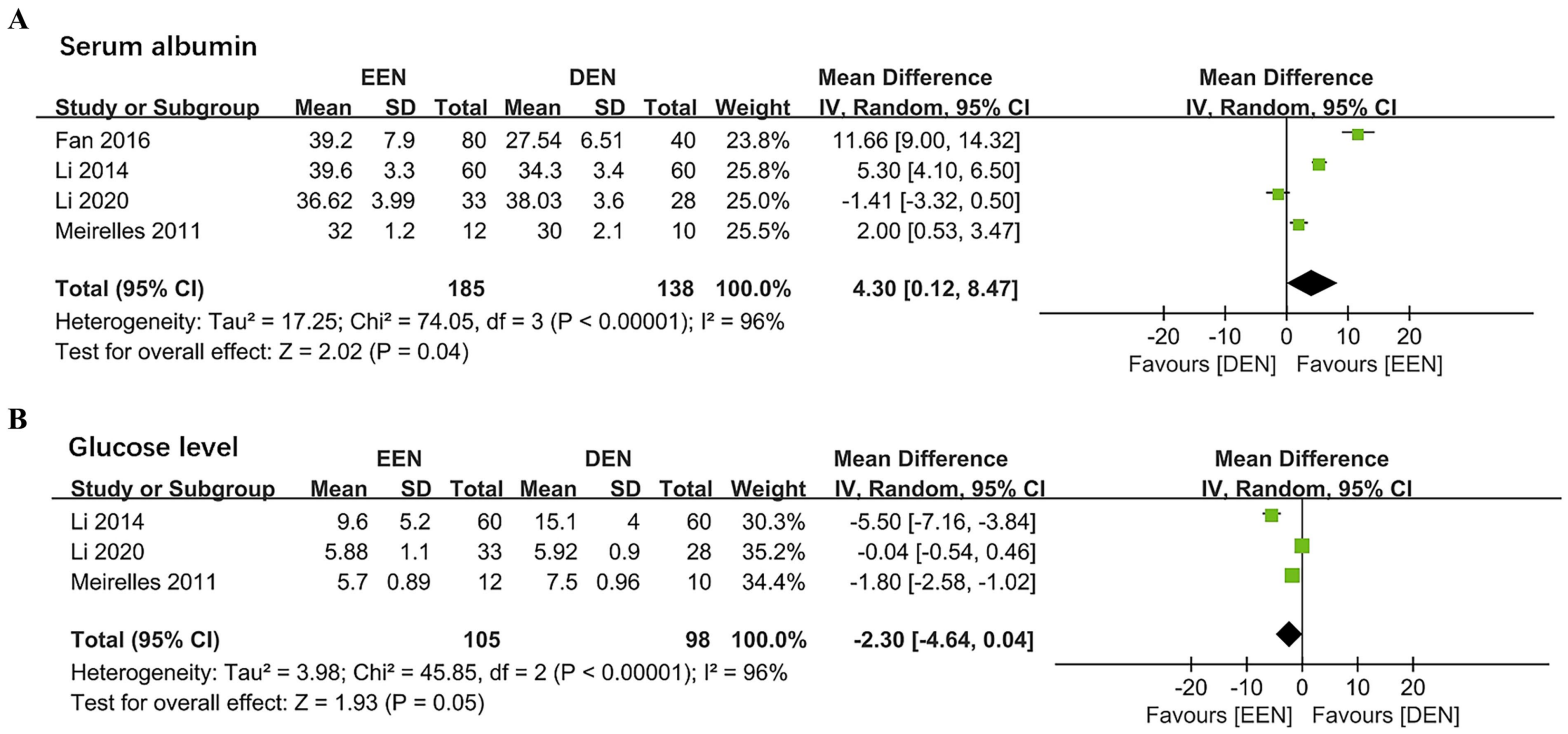
Forest plot of odds ratios for the comparisons between TBI patients with EEN and DEN regarding post-treatment serum albumin levels **(A)** and hospital glucose levels **(B)**. EEN, early enteral nutrition; DEN, delayed enteral nutrition; TBI, traumatic brain injury.

The pooled the results indicated that the EEN significantly increased serum albumin levels (27–30) (MD = 4.30 g/L; 95% CI, 0.12 to 8.47; *p* = 0.04; Figure 4A). After treatment, there was no statistically significant difference in blood glucose levels between the EEN and DEN groups (MD = –2.30 mmol/L; 95% CI, −4.64 to 0.04; *p* = 0.05; Figure 4B) (28–30). For the two secondary outcomes, sensitivity analyses using the simplified transformation method yielded results highly consistent with our primary analysis (Supplemental File 8).

#### Complications

**Figure 5:**
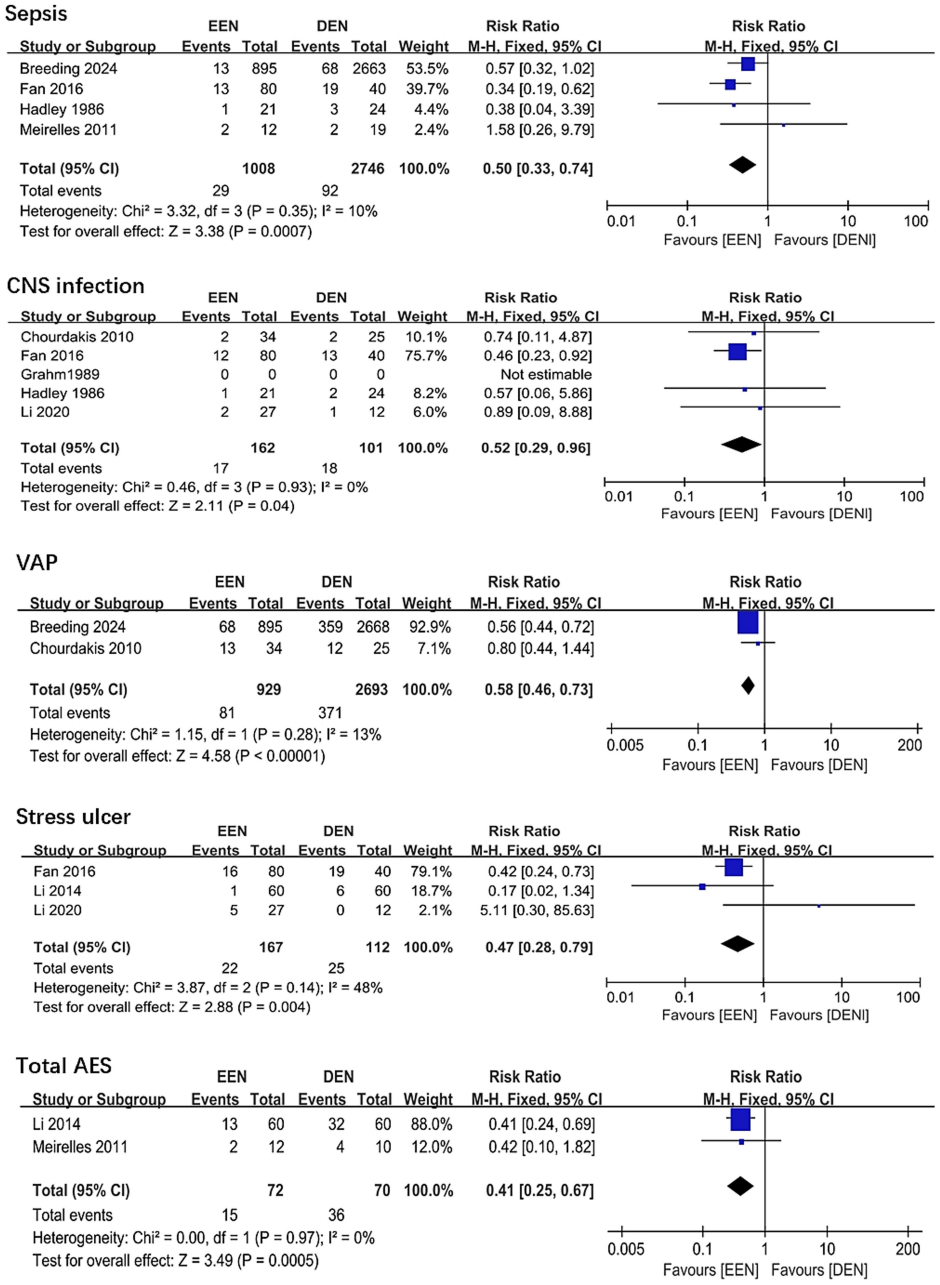
Forest plot of odds ratios for the comparisons of complications in patients with traumatic brain injury received EEN and DEN. EEN, early enteral nutrition; DEN, delayed enteral nutrition; VAP, ventilator associated pneumonia.

**Figure 6:**
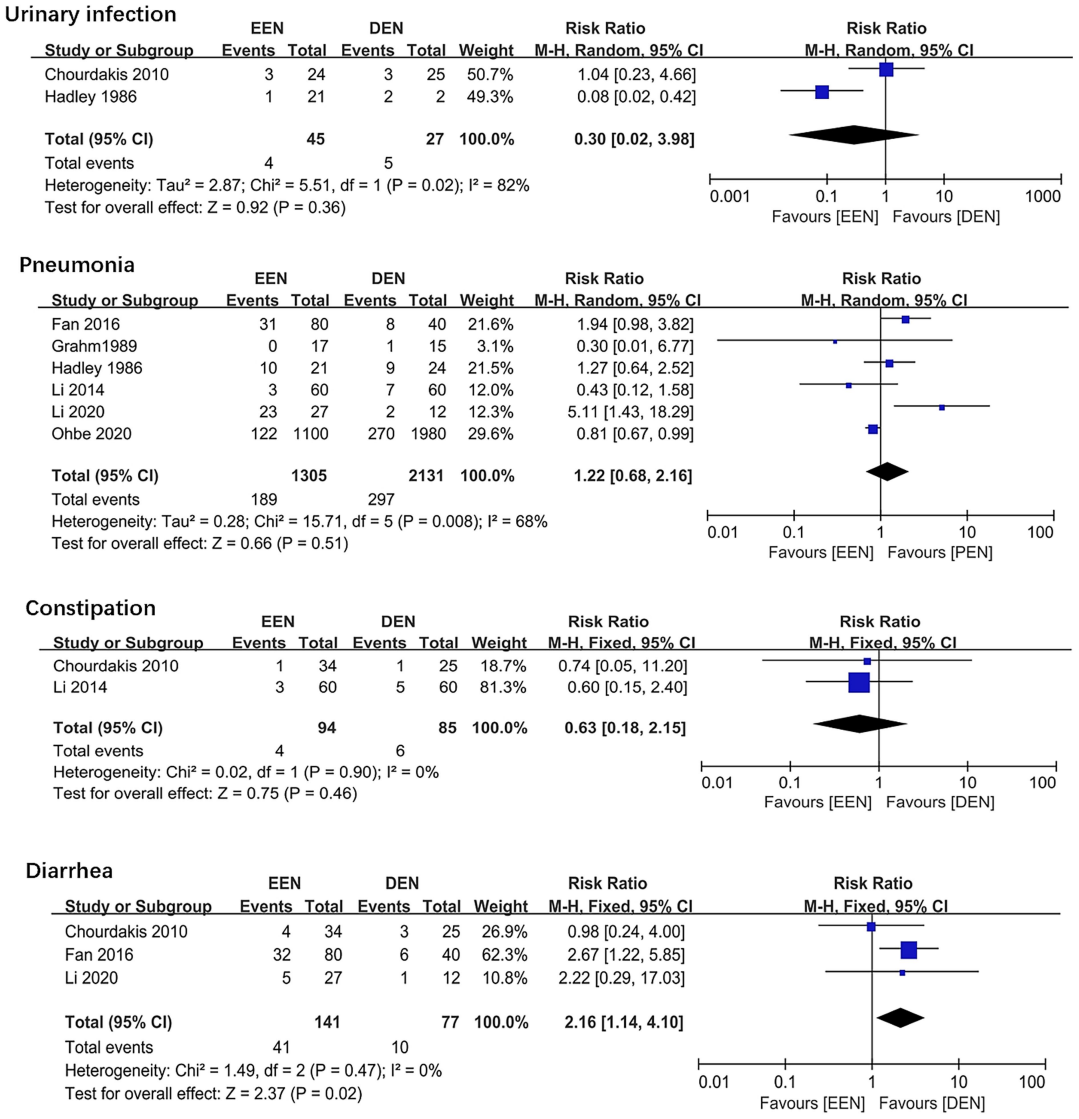
Forest plot of odds ratios for the comparisons of complications in patients with traumatic brain injury received EEN and DEN. EEN, early enteral nutrition; DEN, delayed enteral nutrition; VAP, ventilator associated pneumonia.

Nine studies compared the incidence of various complications between the EEN and DEN groups (10, 11, 17, 18, 27–31) (Supplemental File 9). Pneumonia was the most frequently reported (*n* = 6), followed by central nervous system infection (*n* = 5), sepsis (*n* = 4), diarrhea and stress ulcer (*n* = 3), and ventilator-associated pneumonia (VAP), constipation, total adverse events and urinary infection (*n* = 2). When pooled, EEN showed significantly lower risks of central nervous system infection (RR = 0.52; 95 CI% 0.29–0.96, *p* = 0.04), sepsis (RR = 0.50; 95 CI% 0.33–0.74, *p* = 0.0007), stress ulcer (RR = 0.47; 95 CI% 0.28–0.79, *p* = 0.004), VAP (RR = 0.58; 95 CI% 0.46–0.73, *p* < 0.0001), and total adverse events (RR = 0.41; 95 CI% 0.25–0.67, *p =* 0.0005) (Figure 5). The risks of urinary infections, pneumonia, and constipation were comparable between the two groups (*p* values ranging from 0.36 to 0.51; Figure 6). However, EEN was associated with a higher risk of diarrhea (*p* = 0.02; Figure 6) compared to DEN.

## Discussion

### Summary of findings

This meta-analysis, encompassing 10 studies involving a total of 13,046 patients (10, 11, 17–19, 26–31), evaluates EEN (defined as initiation within 48 h) versus DEN in TBI. While the primary pooled analysis showed no significant reduction in short-term mortality, subgroup analyses revealed that the substantial heterogeneity observed in the primary analysis was largely attributable to variations in study design and calorie intake protocols, with heterogeneity resolved (*I*^2^ = 0%) in RCTs and calorie intake subgroups. Sensitivity analysis restricted to RCTs suggested a potential survival benefit (*p* = 0.03). EEN was associated with improvements in ICU LOS, MV duration and nutritional status, as evidenced by higher serum albumin levels, improved glycemic control, greater caloric support and positive nitrogen balance. EEN also correlated with reduced rates of several complications, albeit with a higher risk of diarrhea.

Collectively, these results suggest a potential clinical benefit of EEN and provide evidence to support and refine current clinical guidelines that advocate for EN in this patient population (15, 16). However, due to the heterogeneity and variable quality of the included studies, the strength of this evidence is limited. These findings should be interpreted as hypothesis-generating, warranting validation through higher-quality trials.

### Comparing with previous studies

Our findings should be interpreted within the context of existing meta-analyses, which have been constrained by substantial methodological heterogeneity. A key limitation across prior work lies in the inconsistent definition of “early” feeding. For instance, an earlier meta-analysis evaluating EN strategies in TBI included studies with widely divergent initiation windows, ranging from 24 h to 7 days post-injury, or relied on subjective criteria such as the return of bowel function (32). This lack of a standardized temporal threshold complicates the interpretation of pooled estimates and limits their applicability to evidence-based clinical protocols. A more recent meta-analysis specifically compared EEN against early PN and delayed EN. However, that analysis primarily focused on a heterogeneous cohort of critically ill patients, with TBI represented only in a subgroup analysis comprising just three small RCTs (16). As a result, it was underpowered to detect meaningful differences in mortality or complication rates in the TBI population.

To address these critical gaps, we adopted a uniform, clinically pragmatic definition of EEN, initiated within 48 h of injury, and integrated recently published studies to conduct a more focused and methodologically rigorous assessment. Our analysis goes beyond mortality to provide a comprehensive evaluation that includes nutritional status and a broad array of clinical complications. The large aggregated sample size, together with consistent and statistically significant results from sensitivity analyses restricted to RCTs, markedly enhances the robustness and generalizability of the observed benefits. Consequently, this meta-analysis serves as a crucial supplement to current guidelines. It not only reinforces existing recommendations favoring EEN in the management of traumatic brain injury but also provides a more nuanced and detailed understanding of its multifaceted clinical impact, thereby supporting more informed, evidence-based decision-making in neurocritical care.

### Mechanisms underlying the clinical benefits of EEN

The clinical benefits of EEN observed in our study can be understood through several interrelated physiological and mechanistic pathways. First, the timing of nutritional intervention is critical. Our strict inclusion criteria, defining EEN as initiation within 48 h of injury, help mitigate a major confounding factor in earlier studies. Many prior studies reporting neutral or adverse effects of EEN actually compared early PN with DEN, where EN was initiated only after clinical tolerance (e.g., return of bowel sounds or gastric motility) was established (33, 34). In contrast, our analysis directly compares feeding strategies based solely on a predefined temporal threshold, thereby offering a more accurate and unbiased assessment of the true physiological and clinical impact of EEN.

Second, the advantages of EEN are strongly supported by both preclinical and clinical pathophysiological evidence. EEN helps preserve gut-associated lymphoid tissue function, maintains mucosal barrier integrity, and reduces bacterial translocation (35, 36), the key mechanisms that attenuate the systemic inflammatory response commonly triggered by TBI. Animal studies suggest that even minimal enteral feeding (e.g., 20% of caloric targets) can help reverse injury-induced dysbiosis and intestinal dysfunction (37). From a clinical standpoint, trophic feeding (typically 10–20 mL/h), even in patients with initial intolerance, may offer essential non-nutritive or pharmacological benefits by stimulating blood flow to the gut (38). This is particularly relevant in mechanically ventilated patients, in whom elevated intrathoracic pressures can impair gut perfusion (39). Moreover, when full caloric targets cannot be met solely via EN during the acute phase, supplemental PN can be employed synergistically (40). EN has also been shown to protect against stress-related gastrointestinal bleeding (41), potentially by attenuating macroscopic ulceration through improved mucosal energy metabolism and stabilization of intramucosal pH (42). Evidence from studies such as that by Fan and colleagues (27) demonstrates that a combined EN + PN strategy enhances both humoral and cellular immunity and more effectively modulates stress responses than PN alone, ultimately translating into improved clinical outcomes.

Third, the significant reduction in nearly all infectious complications with EEN, except for diarrhea, is both clinically significant and biologically plausible. This advantage is intrinsically linked to avoiding prolonged exclusive PN, which is frequently used in DEN protocols. PN is independently associated with increased risks of catheter-related bloodstream infections, hyperglycemia, hepatic steatosis, and gut mucosal atrophy—all of which predispose patients to nosocomial infections (43). By prioritizing the enteral route and minimizing the reliance on PN, EEN directly mitigates these iatrogenic risks, thereby lowering the overall infectious burden.

Fourth, the superior nutritional and metabolic outcomes observed with EEN, including higher serum albumin levels, improved glycemic control, greater caloric intake and positive nitrogen balance, are fundamental to recovery (16). Timely delivery of a complete spectrum of macronutrients and beneficial micronutrients (such as medium-chain triglycerides and fiber for short-chain fatty acid production) supports innate and adaptive immune function, promotes skeletal muscle protein synthesis, facilitates weaning from mechanical ventilation, and encourages earlier participation in rehabilitation (10, 27, 44). These effects collectively create a virtuous cycle: enhanced nutritional status not only increases resilience against complications but also actively drives neurological and functional recovery. This mechanistic cascade provides a compelling explanation for the favorable clinical trends observed in our analysis—and, notably, for the statistically significant mortality benefit demonstrated in the RCT-based sensitivity analysis.

Finally, a notable discrepancy exists between our primary pooled analysis, which showed no significant difference in overall mortality between EEN and DEN strategies, and our pre-specified sensitivity analysis restricted to RCTs, which demonstrated a significant mortality reduction favoring EEN. This divergence likely stems from the inherent differences in study designs included in the meta-analysis. While observational studies contribute valuable real-world data regarding clinical practice patterns, they are susceptible to unmeasured confounding factors, such as variations in injury severity, timing of feeding initiation, and concurrent supportive care protocols that may obscure the true treatment effect. In contrast, RCTs represent the gold standard of evidence by minimizing selection bias and balancing known and unknown confounders through randomization. The significant benefit observed exclusively in the RCT subgroup suggests that the mortality advantage of EEN is robust when evaluated under rigorous experimental conditions. Consequently, although heterogeneous observational data yielded null findings in the aggregate analysis, high-quality RCTs demonstrate a survival benefit of early enteral nutrition in TBI patients.

### Problems and future directions

Our study adopted the 48-h threshold as the defining criterion for EEN, consistent with current major clinical guidelines. However, alternative definitions in the literature contribute to heterogeneity in reported outcomes. For example, Azim et al. defined EEN as initiation within 24 h of injury and reported that patients receiving EEN experienced longer ICU stay (*p* = 0.03), a higher incidence of pneumonia (*p* = 0.04), and greater utilization of hospital resources (45). Conversely, two other studies used a more lenient 72-h window to define EEN and found no significant differences compared with DEN in infectious complications or hospital LOS (20, 46). Moreover, Dhandaplani et al. demonstrated that TBI patients who achieve full enteral feeding within 7 days had significantly better outcomes than those who did so after day 7 (*p* = 0.002) (47).

The discrepancy between these findings and our positive results may be attributed to differences in patient populations, feeding protocols, definitions of “early” nutrition, or outcome measures (32, 48). More fundamentally, they suggest that the relationship between feeding timing and clinical benefit may not be strictly linear. Instead, it likely depends on a constellation of modulating factors—including strategies for managing feeding intolerance, the concomitant use of PN, and the rate and consistency of caloric delivery following initiation (9). Currently, the evidence supporting alternative timeframes, whether stricter (e.g., ≤24 h) or more permissive (e.g., ≤72 h), remains limited and inconsistent, precluding definitive conclusions. Therefore, while our meta-analysis provides robust support for the 48-h window as a clinically effective and pragmatic benchmark, the comparative efficacy of narrower or extended initiation windows warrants further investigation. Future prospective, rigorously designed studies should not only standardize the timing of EN initiation but also harmonize protocols for feeding advancement, tolerance assessment, and monitoring to elucidate the optimal nutritional strategy for patients with TBI.

### Limitations

This meta-analysis has several limitations that warrant consideration. First, the methodological quality of the included RCTs was compromised by insufficient blinding and inadequate allocation concealment. These flaws may have introduced performance and detection bias, potentially leading to an overestimation of the treatment effect of EEN. Second, although we explored sources of heterogeneity via prespecified sensitivity analyses, the primary mortality outcome still exhibited high statistical heterogeneity, suggesting underlying methodological or patient-level differences that may not be fully explained by study design alone. Third, unadjusted confounding factors related to clinical protocols limit our ability to isolate the specific effect of timing. Nutritional pathways and specific feeding protocols varied across studies and could not be fully adjusted for in our analysis, which may have differentially influenced safety and efficacy profiles differently across the included cohorts. Finally, observational studies introduced residual confounding risks, mitigated by emphasizing RCT-only findings. Given the high risk of bias and significant heterogeneity, all pooled results should be interpreted with extreme caution. Future research must prioritize large, multicenter RCTs with rigorous blinding, allocation concealment, and standardized protocols to definitively establish optimal EEN strategies.

## Conclusion

In this meta-analysis, EEN initiated within 48 h post-TBI was associated with a trend toward reduced mortality, shorter ICU LOS, and reduced MV duration compared to DEN. While these findings suggest potential clinical and nutritional benefits, the certainty of the evidence is limited by the high risk of bias and substantial statistical heterogeneity observed in the included studies. Consequently, these results must be interpreted with caution. Future well-designed, large-scale RCTs are warranted to validate these effects and clarify the optimal timing for enteral nutrition in this population.

## Data Availability

The original contributions presented in the study are included in the article/Supplementary material, further inquiries can be directed to the corresponding authors.
