# Supplementary material for: Efficacy of early enteral nutrition in patients with traumatic brain injury: a systematic review and meta-analysis
Source: Front Nutr. 2026 Jul 23;13:1795734. doi: 10.3389/fnut.2026.1795734 (PMC13441933; doi:10.3389/fnut.2026.1795734)
Supplement: Supplementary file 1 [file Data_Sheet_1.DOCX]

**Efficacy of early enteral nutrition in patients traumatic brain injury: a systematic review and meta-analysis**

**Supplemental File**

Supplemental File 1 Search Strategy……………………………………………………………………………………………………………………………………………………………………………………………………………………..…….....…..5

Supplemental File 2 Studies needed for review but not included in the current meta-analysis……………………………………………………………………………………..…………………………………………………...6

Supplemental File 3 Protocols for EEN and DEN across the included studies…………………………………………………………………………………………………………………………………………………………………….7

Supplemental File 4 Quality Assessment of observational studies……… …………………………………………………………………………………………………………………………………………..……………….....…………….9

Supplemental File 5 Quality Assessment of studies……………………..……… …………………………………………………………………………………………………………………………………………..……………….....……….…11

Supplemental File 6 Publication bias……………………. …………….………………………….………………………………………………………………….…………………………………………….....………………………………....……….12

Supplemental File 7 data conversion methods……………………. …………….………………………….…………………………………………………………………………………………..…….....………………………………....……….13

Supplemental File 8 Complications……………………. …………….………………………….………………………………………………………………………………………………………….....…………….…………………....……………….14

**Supplemental File 1**

**Search Strategy**

Database: 4 data bases

Search completed September 25, 2025

----------------------------------------------------------------------------------------------------------------------

**PubMed**

((((traumatic brain injury[Title/Abstract]) OR (craniocerebral injury[Title/Abstract])) OR (head injury[Title/Abstract])) OR (head trauma[Title/Abstract])) AND ((((((enteral nutrition[Title/Abstract]) OR (enteral tube feeding[Title/Abstract])) OR (feeding[Title/Abstract])) OR (parenteral nutrition[Title/Abstract])) OR (gastrointestinal[Title/Abstract])) OR (nutritional support[Title/Abstract]))

**Embase**

No. Query Results

#13. #6 AND #12

#12. #6 OR #7 OR #8 OR #9 OR #10 OR #11

#11. 'gastrointestinal':ab,ti AND [embase]/lim

#10. 'enteral tube feeding':ab,ti AND [embase]/lim

#9. 'feeding':ab,ti AND [embase]/lim

#8. 'feed':ab,ti AND [embase]/lim

#7. 'enteral nutrition':ab,ti AND [embase]/lim

#6. #1 OR #2 OR #3 OR #4 OR #5

#5. 'head trauma':ab,ti AND [embase]/lim

#4. 'head injury':ab,ti AND [embase]/lim

#3. 'traumatic brain injury':ab,ti AND [embase]/lim

#2. 'craniocerebral injury':ab,ti AND [embase]/lim

#1. 'traumatic brain injury'/exp

**Cochrane library**

ID	Search

#1	("traumatic brain injury"):ti,ab,kw (Word variations have been searched)

#2	("craniocerebral injury"):ti,ab,kw (Word variations have been searched)

#3	("head injury"):ti,ab,kw (Word variations have been searched)

#4	("head trauma"):ti,ab,kw (Word variations have been searched)

#5	#1 OR #2 OR #3 OR #4

#6	("enteral nutrition"):ti,ab,kw (Word variations have been searched)

#7	("feed"):ti,ab,kw (Word variations have been searched)

#8	("feeding"):ti,ab,kw (Word variations have been searched)

#9	("gastrointestinal"):ti,ab,kw (Word variations have been searched)

#10 #6 OR #7 OR #8 OR #9

#11 #5 AND #10

**Web of Science**

1. traumatic brain injury [topic]

2. craniocerebral injury [topic]

3. head injury [topic]

4. head injury [topic]

5. or/1-4

6. enteral nutrition [topic]

7. feed[topic]

8. feeding [topic]

9. gastrointestinal [topic]

10. or/6-9

11. 5 and 10

**Supplemental File 2**

**Table. Studies needed for full-reviewed but not included in the current meta-analysis (n=9 trials)**

| No | Study | Reason of exclusion |
| --- | --- | --- |
| 1 | Falcão de Arruda IS, de Aguilar-Nascimento JE. Benefits of early enteral nutrition with glutamine and probiotics in brain injury patients. Clin Sci (Lond). 2004 Mar;106(3):287-92. | No DEN group. |
| 2 | Azim A, Haider AA, Rhee P, Verma K, Windell E, Jokar TO, et al. Early feeds not force feeds: Enteral nutrition in traumatic brain injury. J Trauma Acute Care Surg. 2016 Sep;81(3):520-4. | Defined EEN<24 hour of TBI |
| 3 | Borzotta AP, Pennings J, Papasadero B, Paxton J, Mardesic S, Borzotta R, et al. Enteral versus parenteral nutrition after severe closed head injury. J Trauma. 1994 Sep;37(3):459-68. | Defined EEN<72 hour of TBI |
| 4 | Minard G, Kudsk KA, Melton S, Patton JH, Tolley EA. Early versus delayed feeding with an immune-enhancing diet in patients with severe head injuries. JPEN J Parenter Enteral Nutr. 2000 May-Jun;24(3):145-9. | Defined EEN<60 hour of TBI |
| 5 | Suchner U, Senftleben U, Eckart T, Scholz MR, Beck K, Murr R, et al. Enteral versus parenteral nutrition: effects on gastrointestinal function and metabolism. Nutrition. 1996 Jan;12(1):13-22. | Included not TBI patients |
| 6 | Rapp RP, Young B, Twyman D, Bivins BA, Haack D, Tibbs PA, et al. The favorable effect of early parenteral feeding on survival in head-injured patients. J Neurosurg. 1983 Jun;58(6):906-12. | Defined EEN as recovery of intestinal tolerance |
| 7 | Young B, Ott L, Twyman D, Norton J, Rapp R, Tibbs P, et al. The effect of nutritional support on outcome from severe head injury. J Neurosurg. 1987 Nov;67(5):668-76. | Defined EEN as recovery of intestinal tolerance |
| 8 | Nataloni S, Gentili P, Marini B, Guidi A, Marconi P, Busco F, et al Nutritional assessment in head injured patients through the study of rapid turnover visceral proteins. Clin Nutr. 1999 Aug;18(4):247-51. | Patients received EN after 48 hours of TBI |
| 9 | Härtl R, Gerber LM, Ni Q, Ghajar J. Effect of early nutrition on deaths due to severe traumatic brain injury. J Neurosurg. 2008 Jul;109(1):50-6. | Nutrition start <5 days vs >5 days |
| 10 | Lepelletier D, Roquilly A, Demeure dit latte D, Mahe PJ, Loutrel O, Champin P, et al. Retrospective analysis of the risk factors and pathogens associated with early-onset ventilator-associated pneumonia in surgical-ICU head-trauma patients. J Neurosurg Anesthesiol. 2010 Jan;22(1):32-7. | Compared EN >2000 kcal vs <2000 kcal before day 5 as a risk factor of VAP |
| 11 | Dhandapani S, Dhandapani M, Agarwal M, Chutani AM, Subbiah V, Sharma BS, Mahapatra AK (2012) The prognostic significance of the timing of total enteral feeding in traumatic brain injury. Surg Neurol Int 3:31. | EEN in all “as soon as possible” |
| 12 | Vitaz TW, Jenks J, Raque GH, Shields CB. Outcome following moderate traumatic brain injury. Surg Neurol. 2003 Oct;60(4):285-91; discussion 291. | Defined EEN<4 days of TBI |

**Supplemental File 3: Quality Assessment of observational studies**

| **Reference** | **TBI Severity Definition** | **Nutritional Route** | **Feeding Dose & Protocol Details** |
| --- | --- | --- | --- |
| **Breeding 2024** | • **Population:** Adults (≥18 y), moderate-severe isolated blunt TBI. • **GCS:** Moderate (9–12), Severe (≤8). • **AIS:** Head AIS ≥3. | **Enteral Nutrition (EF)** • Identified via ICD procedural codes (e.g., nasogastric/jejunal tubes). • Specific tube type not differentiated. | • **Timing Stratification:** Ultra-early (<6 h), Early (6–24 h), Intermediate (24–48 h), Late (>48 h). • **Dosing:** Specific caloric or protein targets not reported; primary endpoints were ICU-LOS and complications. |
| **Chiang 2012** | • **Population:** Severe TBI (sTBI). • **GCS:** 4–8 (excluded GCS ≤3). • **Comparison:** GCS 4–5 vs. 6–8. | • **EN Group:** Enteral nutrition (route unspecified). • **Non-EN Group:** IV fluids only (no EN/TPN). | • **Initiation:** EN initiated within 48 h post-injury. • **Dose:** Initial 500 kcal/day, gradually increased to estimated requirements; Formula: 52.9% CHO, 31.1% Fat, 16.4% Protein. • **Non-EN:** Maintenance fluids only for 7 days. |
| **Chourdakis 2010** | • **GCS:** Actual enrollment 5–6 (Severe). • **Exclusion:** GCS ≥9. • **APACHE II:** ~15.7. | **Enteral Nutrition (EN)** • **Access:** Nasogastric tube. • **Groups:** **Early** (24–48 h) vs. **Delayed** (>48 h). | • **Energy Target:** Based on indirect calorimetry (REE), aiming for 100% of requirements (~1792–1862 kcal/d). • **Protein Target:** 1.5 g/kg body weight. • **Infusion:** Started at 30 mL/h, increased to 80–100 mL/h within 48h. |
| **Fan 2016** | • **GCS:** 6–8 (Severe sTBI). • **NRS 2002:** ≥3 (high risk). | • **EN:** Nasogastric tube (pump). • **PN:** Central vein. **Combined (EN+PN):** NG + Central vein. | • **Energy Target:** 25–30 kcal/kg/d (105–126 kJ/kg/d). • **EN Protocol:** Max 1500 mL/d, gradual increase over 7 days, pump speed <75 mL/h (1.5 kcal/mL). • **EN+PN:** EN max 1000 mL/d (<50 mL/h), remainder supplemented by PN. |
| **Grahm 1989** | • **GCS:** <10 (Mean: 5.1 vs. 7.1, Severe). • **Population:** Admitted within 6h. | • **Experimental:** Early Nasojejunal feeding. • **Control:** Delayed Gastric feeding. | • **Energy Target:** Matched daily measured REE (~2535 kcal/d); Experimental group achieved 2102 kcal/d. • **Nitrogen/Protein:** Exp: 11.1 g N/d (~69 g protein); Control: 5.6 g N/d. • **Formula:** Vital HN (1 kcal/mL), adjusted for concentration (1.0–2.0 kcal/mL). |
| **Hadley 1986** | • **GCS:** ≤10 (Mean 5.8, Severe). • **Population:** Isolated head injury, few with minor polytrauma. | • **TPN Group:** Total Parenteral Nutrition (Central line). • **NG Group:** Enteral Nutrition (Nasogastric tube). | • **Energy Target:** 140% of predicted BMR via indirect calorimetry/Harris-Benedict (~146% of measured REE). • **Nitrogen:** TPN 13.1 g N/d vs. NG 11.4 g N/d. • **Duration:** 14 days or until urine nitrogen <14 g/d. |
| **Li 2014** | **Population:** Post-operative head injury patients. **NRS 2002:** ≥3. | • **EN Group:** Nasogastric tube (post-op 48 h). • **PN Group:** Central vein (post-op 48 h, constant rate; switched to NG after 7–8 days). | • **EN Protocol:** Initial 45 g fiber formula + 170 mL water (200 mL total), 6–7 times/day; Formula: 412 kcal/100g, 18g protein/100g. • **PN Protocol:** Glucose, lipid, amino acids; non-protein calorie ratio matched EN, Cal:N ratio 155:1. |
| **Li 2020** | • **GCS:** 3–15 (Mean 7.92); stratified ≤8 vs. ≥9. • **NRS 2002:** All ≥3. | • **Control:** PN alone (IV). • **Observation:** PN + EN (within 48 h post-op). | • **EN Method:** Nasogastric tube for unable patients; Oral Nutritional Supplements (ONS) for those with inadequate intake. • **Dosing:** Specific kcal/kg or g protein/kg targets not reported; described as "adequate support." Authors noted targets were not met. |
| **Meirelles 2011** | • **GCS:** 9–12 (Moderate TBI). • **Population:** 18–60 y, CT-confirmed, hemodynamically stable. | • **EN Group (n=12):** Naso/orogastric tube (pump). • **TPN Group (n=10):** Total Parenteral Nutrition (Central line). | • **Energy Target:** 25–30 kcal/kg/day. • **Protein Target:** 1.5 g/kg/day (casein added to EN formula). • **Implementation:** Targets planned to be reached within 3 days; progressive caloric deficit occurred in both groups. |
| **Ohbe 2020** | • **GCS:** ≤8 (Severe TBI, corresponding to Japan Coma Scale ≥30). • **Population:** Nationwide inpatient database (DPC), adults with intracranial injury. | **Enteral Nutrition (EN) Only** • **Early EN:** Initiated ≤2 days post-admission. • **Delayed EN:** Initiated 3–5 days post-admission. | • **Dosing Details:** Specific target kcal/kg or g protein/kg not reported in the methods/outcomes. • **Outcomes:** Early EN associated with lower pneumonia rate and shorter LOS, but no mortality difference. |
| **Suchner 1996** | • **GCS:** ≤10 (Severe TBI or spontaneous lesions requiring craniotomy). • **Baseline Mean:** EN 8.0±2.0, TPN 7.5±2.1. | • **TEN Group:** Total Enteral (Nasogastric, Osmolite® 1 kcal/mL). • **TPN Group:** Total Parenteral (Central line). | • **Energy Basis:** Daily indirect calorimetry (REE), target = REE (no stress factor). • **Titration:** EN started at 20 mL/h, target by Day 5; TPN started at 50% REE (Day 1), 100% REE from Day 3. • **Macronutrients:** 16.7% Protein, 30% Fat, 53.3% CHO (matched between groups). |

**Supplemental File 4: Quality Assessment of Observational Studies**

| **Study (First Author, Year)** | **Domain** | **Item Description** | **Score (Stars)** | **Notes/Rationale** |
| --- | --- | --- | --- | --- |
| Chiang et al. (2012) | Selection | Representativeness of the exposed cohort / Selection of the non-exposed cohort | ★★★ | Both cohorts were selected from 18 hospitals; matching on demographics. |
|  |  | Ascertainment of exposure | ★ | Confirmed via medical records. |
|  | Comparability | Control for confounding (Age, GCS, etc.) | ★★★ | Adjusted for age, gender, initial GCS, and recruitment period. |
|  | Outcome | Assessment of outcome / Follow-up | ★ | Outcome (mortality/GCS) confirmed via records; follow-up period defined. |
|  |  | ***Total*** | ***8/9*** | ***High Quality*** |
| Ohbe et al. (2020) | Selection | Representativeness of the exposed cohort / Selection of the non-exposed cohort | ★★★ | Nationwide database (large sample); propensity score matching performed. |
|  |  | Ascertainment of exposure | ★ | Exposure (EN timing) ascertained from administrative claims. |
|  | Comparability | Control for confounding (PSM) | ★★★ | Propensity score matching for age, sex, BMI, comorbidities, and injury severity. |
|  | Outcome | Assessment of outcome / Follow-up | ★★★ | Complete follow-up (in-hospital); outcome definition validated. |
|  |  | ***Total*** | ***8/9*** | ***High Quality*** |
| Li et al. (2020) | Selection | Representativeness of the exposed cohort / Selection of the non-exposed cohort | ★★★ | Single-center retrospective; non-exposed group selected from same hospital. |
|  |  | Ascertainment of exposure | ★ | Exposure (PN vs PN+EN) confirmed via records. |
|  | Comparability | Control for confounding | ★ | Basic adjustment (Score 1) - Controlled for basic demographics but lacked full severity adjustment. |
|  | Outcome | Assessment of outcome / Follow-up | ★ | Adequate follow-up (discharge/death); outcome assessment via records. |
|  |  | ***Total*** | ***6/9*** | ***Moderate Quality*** |
| Breeding et al. (2024) | Selection | Representativeness of the exposed cohort / Selection of the non-exposed cohort | ★★★ | National database (ACS TQP); representativeness is high. |
|  |  | Ascertainment of exposure | ★ | Exposure (timing) ascertained via ICD procedure codes. |
|  | Comparability | Control for confounding | ★ | Controlled for demographics and facility level; lacked detailed clinical data (sedation/ICP). |
|  | Outcome | Assessment of outcome / Follow-up | ★★★ | Complete follow-up (in-hospital outcomes); standardized definitions. |
|  |  | ***Total*** | ***8/9*** | ***High Quality*** |

**Scoring System**: The NOS scale uses a star system (max 9 stars). Selection (4 stars), Comparability (2 stars), Outcome (3 stars).

**Quality Grading**: High Quality (8-9 stars), Moderate Quality (6-7 stars), Low Quality (<6 stars).

**Supplemental File 5: Quality Assessment of observational studies**

**Risk of bias summary: review authors' judgements about each risk of bias item for each included study.**

**Risk of bias graph: review authors' judgements about each risk of bias item presented as percentages across all included studies.**

**Supplemental File 6: Funnel plot of comparison of mortality of all studies.**

No significant funnel plot asymmetry was detected (p = 0.703), suggesting a low risk of publication bias.

**Supplemental File 7: data conversion methods and sensitivity analyses.**

(1) Data conversion methods （give examples）

| Sample size | Method | Formula | Median | QI | Q2 | Mean | SD |
| --- | --- | --- | --- | --- | --- | --- | --- |
| 3080 | Primary analysis | Mean =(Q1+Median+Q3)/3; Sd = (Q3-Q1)/1.35 | 38 | 23 | 55 | **38.7** | **23.7** |
| 3080 | Sensitivity analysis | Mean = Median; Sd = (Q3-Q1)/1.35 | 38 | 23 | 55 | **38** | **23.7** |
| 22 | Primary analysis | Mean =(Q1+Median+Q3)/3; Sd = (Q3-Q1)/3.3 | 1.5 | 1.4 | 1.75 | **1.55** | **0.26** |
| 22 | Sensitivity analysis | Mean = Median; Sd = (Q3-Q1)/1.35 | 1.5 |  |  | **1.5** | **0.11** |

（2）Only data from Ohbe et al. (2020) regarding hospital LOS and cost required transformation. Sensitivity analyses confirmed that the simplified method produced results highly consistent with our primary findings.

Forest plot of odds ratios for the comparisons of hospital length of stay between patients with TBI who received EEN and DEN **in sensitivity analysis**. EEN = early enteral nutrition; DEN = delayed enteral nutrition.

Forest plot of odds ratios for the comparisons of cost between patients with TBI who received EEN and DEN **in sensitivity analysis**. EEN = early enteral nutrition; DEN = delayed enteral nutrition.

**Supplemental File 8**

**Table: Summarized Complications among he included studies.**

| Complications | Breeding 2024 | | Chourdakis 2010 | | Fan 2016 | | HadIey 1986 | | Li 2014 | | Li 2020 | | MeireIIes 2011 | | Ohbe 2020 | | Grahm1989 | |
| --- | --- | --- | --- | --- | --- | --- | --- | --- | --- | --- | --- | --- | --- | --- | --- | --- | --- | --- |
|  | **EEN** | **DEN** | **EEN** | **DEN** | **EEN** | **DEN** | **EEN** | **DEN** | **EEN** | **DEN** | **EEN** | **DEN** | **EEN** | **DEN** | **EEN** | **DEN** | **EEN** | **DEN** |
| Total patients | **3563** | **2668** | **34** | **25** | **80** | **40** | **21** | **24** | **60** | **60** | **33** | **28** | **12** | **10** | **1100** | **1980** | **17** | **15** |
| ARDS | 13 | 86 |  |  |  |  |  |  |  |  |  |  |  |  |  |  |  |  |
| Sepsis | 13 | 68 |  |  | 13 | 19 | 1 | 3 |  |  |  |  | 2 | 2 |  |  |  |  |
| VAP | 48 | 359 | 13 | 12 |  |  |  |  |  |  |  |  |  |  |  |  |  |  |
| Pneumonia |  |  |  |  | 31 | 8 | 10 | 9 | 3 | 7 | 24 | 4 |  |  | 122 | 270 | 2 | 3 |
| CNS infection |  |  | 2 | 2 | 12 | 13 | 1 | 2 |  |  | 7 | 1 |  |  |  |  | 0 | 1 |
| Stress ulcer |  |  |  |  | 16 | 19 |  |  | 1 | 6 | 5 | 0 |  |  |  |  |  |  |
| Diarrhea |  |  | 4 | 3 | 32 | 6 |  |  |  |  | 5 | 1 |  |  |  |  |  |  |
| Bacteremia |  |  | 3 | 4 |  |  |  |  |  |  |  |  |  |  |  |  |  |  |
| Hyperglycemia |  |  | 5 | 4 |  |  |  |  |  |  |  |  |  |  |  |  |  |  |
| Feeding intolerance |  |  | 3 | 3 |  |  |  |  |  |  |  |  |  |  |  |  |  |  |
| Constipation |  |  | 1 | 1 |  |  |  |  | 3 | 5 |  |  |  |  |  |  |  |  |
| Urinary infection |  |  | 3 | 3 |  |  | 1 | 2 |  |  |  |  |  |  |  |  |  |  |
| Hypoproteinemia |  |  |  |  | 29 | 32 |  |  |  |  |  |  |  |  |  |  |  |  |
| Wound infection |  |  |  |  |  |  | 2 | 1 |  |  |  |  |  |  |  |  |  |  |
| Total AES |  |  |  |  |  |  |  |  | 13 | 32 |  |  | 0 | 2 |  |  |  |  |
| Renal failure |  |  |  |  |  |  |  |  | 2 | 4 |  |  |  |  |  |  |  |  |
| Heart failure |  |  |  |  |  |  |  |  | 2 | 5 |  |  |  |  |  |  |  |  |
| Pressure sores |  |  |  |  |  |  |  |  | 2 | 5 |  |  |  |  |  |  |  |  |

AES = adverse events; ARDS= acute respiratory distress syndrome; CNS = central nervous system; DEN = delayed enteral nutrition; EEN= early enteral nutrition; VAP = ventilation-associated pneumonia.
